# Vision-assisted micromanipulation using closed-loop actuation of multiple microrobots

**DOI:** 10.1186/s40638-017-0064-4

**Published:** 2017-10-30

**Authors:** M. Arifur Rahman, Noboru Takahashi, Kawai F. Siliga, Nigel K. Ng, Zhidong Wang, Aaron T. Ohta

**Affiliations:** 1 Department of Electrical Engineering, University of Hawaii at Manoa, 2540 Dole Street, Holmes Hall 483, Honolulu, HI 96822 USA; 2 Department of Advanced Robotics, Chiba Institute of Technology, 2-17-1 Tsudanuma, Narashino, Chiba 275-0016 Japan

**Keywords:** Microrobot, Opto-thermocapillary flow, Micromanipulation, Closed-loop control, Grasp planning, Path planning, Caging

## Abstract

Accurate control and precise positioning of opto-thermocapillary flow-addressed bubble microrobots are necessary for micromanipulation. In addition, micromanipulation using the simultaneous actuation of multiple microrobots requires a robust control system to enable independent motion. This paper demonstrates a hybrid closed-loop vision-assisted control system capable of actuating multiple microrobots simultaneously and positioning them at precise locations relative to micro-objects under manipulation. A vision-assisted grasp-planning application was developed and used to calculate the necessary trajectories of the microrobots to form cages around micro-objects. The location of the microrobots and the micro-objects was detected at the caging locations using a particle-tracking application that used image feedback for precise positioning. The closed-loop image feedback information enabled the position update of the microrobots, allowing them to precisely follow the trajectory and caging locations calculated by the grasp-planning application. Four microrobots were assigned to cage a star-shaped micro-object using the closed-loop control system. Once caged, the micro-object was transported to a location within the workspace and uncaged, demonstrating the micromanipulation task. This microrobotic system is well suited for the micromanipulation of single cells.

**Electronic supplementary material:**

The online version of this article (doi:10.1186/s40638-017-0064-4) contains supplementary material, which is available to authorized users.

## Background

Microrobots in a liquid medium are an efficient tool for bio-micromanipulation. They have broad applications in biological engineering [1–4], biomedical engineering [5, 6], and tissue engineering [7, 8]. Microrobots, which are untethered submillimeter actuators, have been utilized for the transportation of micro-objects including living cells [5, 7, 9], with micron to submicron resolution [5, 7, 9–11]. Microrobot-assisted bio-micromanipulation is capable of cell separation and sorting [10, 12, 13], cell trapping, isolation, and transport [12, 14], cell-laden hydrogel assembly [7], and cell lysis and molecular delivery [15]. Microrobots can be actuated using various actuation methods including magnetic actuation [8, 9, 16–20], bacterial propulsion [3, 4], biomimetic propulsion [21, 22], and opto-thermal actuation [7, 12, 13, 15]. However, the degree of control required and achieved by the various methods of actuation depends upon the actuation force and the number of microrobots being addressed independently.

One significant challenge to controlling many microrobots independently is the use of a global actuation force, as employed by magnetic microrobots [9, 23] and bio-inspired magnetic swimming microrobots [6, 24]. Despite the challenges, the independent actuation of multiple magnetic microrobots has been demonstrated and was made possible by fabricating the microrobots with different dimensions to obtain different magnetic signatures [25, 26]. Bacteria-propelled microrobots based on the motility of the bacteria have a limited degree of controllability [27], but the use of electrical signals [28], UV light [4], or chemical energy [3, 27] in conjunction with bacteria propulsion helps to achieve higher controllability, and has been used to actuate multiple microrobots [3]. Bacteria-inspired microrobots actuated by magnetic [29], acoustic [30], or a combination of these forces [31] is capable of parallel control, but complex motion, such as actuation along multiple trajectories, is more difficult [32].

One type of microrobot that allows the parallel actuation of many microrobots independently is the opto-thermocapillary flow-addressed bubble (OFB) microrobot [12]. This microrobot consists of a gas bubble in a liquid medium and is actuated by opto-thermal forces [33]. Parallel actuation of many OFB microrobots allows higher-throughput micromanipulation [34] and cooperative manipulation [35]. However, the simultaneous actuation of many microrobots is beyond the capacity of a human operator using a manual control interface, such as a mouse [35] or touch screen [36]. An automated, closed-loop control system is required to control many microrobots at the same time.

In previous work, a small number of OFB microrobots performed microassembly [35–37], single-cell assembly [12], cell-laden hydrogel assembly [7], and single-cell poration [15]. These applications require microscale accuracy under optical microscopy. As an example, cell-laden hydrogel structures need to be positioned in contact for cell culturing. A closed-loop automated control system benefits the above micromanipulation tasks by enabling increased throughput, cooperative microassembly, and the automated planning and execution of assembly tasks.

Individual and independent control of many OFB microrobots in parallel requires automated and sophisticated control, including features such as grasp planning for caging, path planning for obstacle avoidance, and moving along the shortest path to a destination. Moreover, when multiple OFB microrobots are simultaneously actuated, precise positioning and actuation, as well as knowledge of the payload location, will enhance the accuracy and resolution of the micromanipulation.

In this work, a hybrid closed-loop control system for an OFB microrobot system was developed. The hybrid system uses an open-loop computer-generated holographic control system (developed in LabVIEW) to generate the optical patterns necessary to actuate multiple OFB microrobots simultaneously. The closed-loop part of the hybrid system includes an image-processing algorithm (developed in MATLAB) that provides image feedback control; this allows the actuation of multiple OFB microrobots and the knowledge of the locations of the objects under manipulation. The control system also includes a grasp-planning algorithm (developed in MATLAB) that determines the shortest path and suitable grasping point of micro-objects. Finally, the closed-loop automatic actuation of four microrobots was demonstrated; the OFB microrobots cooperatively caged a star-shaped SU-8 microstructure and transported it to a desired location within the workspace. The hybrid control system achieved higher accuracy compared to open-loop actuation.

## Methods

**Fig. 1:**
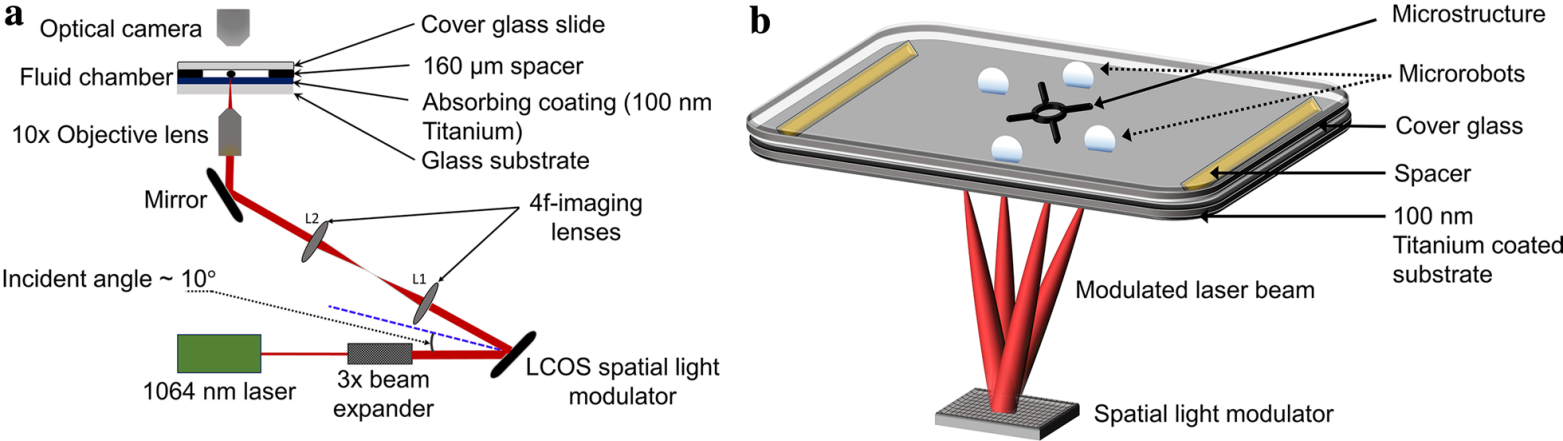
Opto-thermocapillary flow-addressed bubble (OFB) microrobot generation and actuation setup. **a** Experimental setup for OFB microrobot micromanipulation. **b** A schematic diagram showing a single laser beam modulated by the spatial light modulator, creating a user-designed pattern on the substrate. Each optical spot on the substrate can generate and actuate an OFB microrobot around the micro-object under manipulation, enabling parallel control of multiple entities

A 1064-nm Nd:YAG, single-mode (TEM00) linearly polarized laser (Laser Quantum, Ventus 1064, 1.5 + W) with a 2-mm beam diameter was expanded using a 3x  beam expander (Fig. 1a). The expanded beam was incident at approximately 10 degrees on a spatial light modulator (SLM, Hamamatsu, LCOS-SLM X10468-07) with an active area of 16 mm by 12 mm. The collimated laser beam was modulated by the SLM, which displayed an 800 pixel by 600-pixel computer-generated hologram (CGH) created using a modified version of the Red Tweezers program [38]. The modulated wavefront containing the user-defined pattern was 4-*f* imaged by placing lens L1 at its focal length from the SLM, and lens L2 at its focal length away from the Fourier plane. A 0.42-N.A. 10X objective lens (Mitutoyo) was placed at the focal length of lens L2, focusing the optical pattern on the light-absorbing coating of the substrate. The 1-mm-thick glass substrate is coated with 100 nm of titanium and formed a fluidic chamber when bonded to a 1-mm-thick glass slide with a spacer layer consisting of 160-μm double-sided polyimide tape. The liquid medium used in the fluidic chamber was silicone oil (Fisher Scientific, S159-500). A 1600 pixel by 1200 pixel camera (Point Grey Flea3) was used for image capture. One pixel in the image corresponds to 1.02 μm on the workspace.

The spatial light modulator creates the optical pattern desired by the user (Fig. 1b). Each optical spot that is focused on the light-absorbing layer of the substrate produces a localized hot spot, vaporizing a small volume of the liquid, and thus generating an OFB microrobot [28]. The thermocapillary flow generated by the temperature gradient and resulting surface tension gradient at the gas–liquid interface of the bubble pulls the OFB microrobot toward the center of the localized hot spot [28].

## Vision-assisted closed-loop control system

**Fig. 2:**
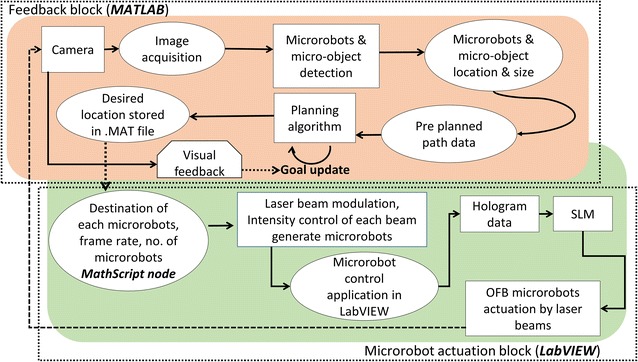
Block diagram of the hybrid vision-assisted closed-loop control system and the microrobot actuation system. The feedback block determines the positions of the microrobots by analyzing the video from the microscope camera and generates trajectories for the microrobots. The microrobot actuation block creates the appropriate optical patterns to move the microrobots

The hybrid closed-loop control system developed in this work has two different parts: the microrobot actuation block developed in LabVIEW, and the image-processing and grasp-planning block developed in MATLAB. The data transfer between the LabVIEW and the MATLAB control blocks was accomplished using the MathScript module of LabVIEW. Figure 2 shows the block diagram of the complete control system. The actuation of microrobots was accomplished by the “microrobot actuation block.” An open-loop computer-generated holographic (CGH) control system was developed in LabVIEW [33]. An optical spot is represented by a circular spot on the LabVIEW user interface and is maneuvered using manual or automatic control. The user enters the target location of each microrobot, and the MathScript module calculates the navigation parameters, such as initial location, destination, frame rate, and frame size, and passes them to the control application. The control application in LabVIEW then sends the data to the OpenGL Shader hologram engine, which calculates the corresponding hologram using the direct superposition algorithm. The holograms corresponding to the optical pattern are then displayed on the SLM.

The feedback block was developed in MATLAB (Fig. 2). The major function of the feedback loop is to process the camera image of the workspace and output data on the OFB microrobots and the micro-objects under manipulation. The captured image was analyzed by the Hough transform algorithm, providing the microrobot’s locations and sizes, and the micro-object’s locations and sizes. The Hough transform detected the shapes in the image, which were then matched to prestored object data. The image data were converted to grayscale and then to a binary image using Otsu thresholding [39] to make the image suitable for the Hough transform. Detection of unwanted objects, such as the white circular spot at the center of Microrobot 1 in Fig. 4a, was reduced by defining the size ranges of the objects to detect. A grasp-planning algorithm was also developed in MATLAB to plan the course, speed, and other microassembly-related parameters for efficient manipulation. Once the microrobot data and micro-object data are available in the MATLAB workspace, the grasp-planning module utilizes user-desired preplanned conditions to calculate the final location of each microrobot for accurate grasping. Once the final destination of each microrobot is calculated, it is saved in a.mat file for subsequent use by the microrobot actuation block. During initiation of the actuation, the destination data from the .mat file are loaded into the MathScript module of LabVIEW.

## Results

The hybrid control system described above was used to perform closed-loop actuation of a single microrobot, grasp planning of multiple microrobots for a micromanipulation task, closed-loop actuation of multiple microrobots, and open-loop micromanipulation of a star-shaped SU-8 microstructure.

### Closed-loop actuation of a single microrobot

**Fig. 3:**
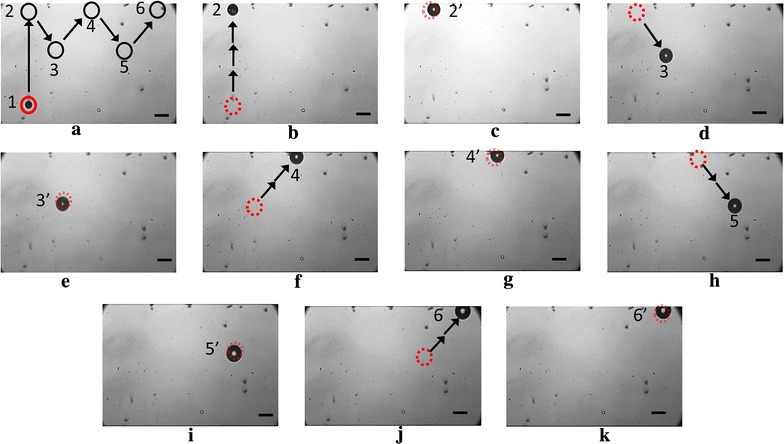
An OFB microrobot was actuated using open-loop control from waypoint 1 to 2 (**a**, **b**), waypoint 2′ to 3 (**c**, **d**), waypoint 4′ to 5 (**g**, **h**), and waypoint 5′ to 6 (**i**, **j**), at velocities of 70, 43, 44, 46, and 39 μm/s, respectively. The OFB microrobot location was updated by the vision-assisted closed-loop control at waypoints 2 to 6 (**c**, **e**, **g**, **i**, **k**). The dotted circles mark the previous location of the microrobot. The average size of the microrobot is approximately 85 μm over the duration of the actuation. The scale bar is 100 μm

One of the benefits of the closed-loop control of OFB microrobots is the ability to accurately update the position of the microrobot using data from the image-processing algorithm. Here, we have demonstrated actuation of one microrobot along a preset zigzag path using the hybrid control system (Fig. 3). The microrobot was actuated from its initial location to 5 waypoints (waypoints 2–6 in Fig. 3) using automated open-loop control sequences. Each point-to-point actuation (1–2, 2–3, 3–4, 4–5, and 5–6; see Fig. 3a) consisted of a series of 30 smaller actuation segments, at a rate of 2 Hz. The actuation velocity between the waypoints varied.

First, the microrobot was actuated from position 1–2 using open-loop control, a distance of 1048 μm (Fig. 3a, b). At location 2 (Fig. 3b), the feedback block captured an image of the workspace, detected the physical bubble location within the workspace, and compared it with the intended destination set in the LabVIEW user interface. The microrobot was then moved to a new location (2′ in Fig. 3c) to correct for the positioning error. Similarly, the microrobot position was determined by the feedback block at each waypoint (3–6 in Fig. 3d, f, h, j), compared with the preset destination in LabVIEW actuation block, and then moved to minimize the difference between the preset destination and the actual position (3′, 4′, 5′, 6′ in Fig. 3e, g, i, k). The position error calculated by the feedback block at 2′, 3′, 4′, 5′, 6′ in Fig. 3 was 5.25, 14.1, 5.1, 16.5, and 2.8 μm, respectively. The position error was then reduced to approximately 1 µm after the microrobot locations were updated using the hybrid closed-loop control system.

### Grasp planning

Path planning refers to determining a collision-free path for a moving object among obstacles [18]. In this work, a grasp-planning algorithm determines the geometry and location of a microrobot and a micro-object payload. The output of the grasp-planning algorithm is sequences of microrobot locations that form a trajectory from its initial position to its goal position, which is in reference to the payload. There were no obstacles present in the workspace when caging the payload, but the algorithm is capable of determining collision-free path about obstacles. (Additional file 1: Figure S4). The grasp-planning algorithm was used to create a cage of four OFB microrobots around a star-shaped SU-8 microstructure, and transport the object to another location. The star-shaped microstructure consisted of four arms, each 59 μm in length, and a hollow circular center with an inner diameter of 62 μm. The width of the wall around the circular center and the width of the arms were approximately 66 μm. The thickness of the SU-8 was 50 μm, and the structure had an approximate mass of 2.35 μg.

**Fig. 4:**
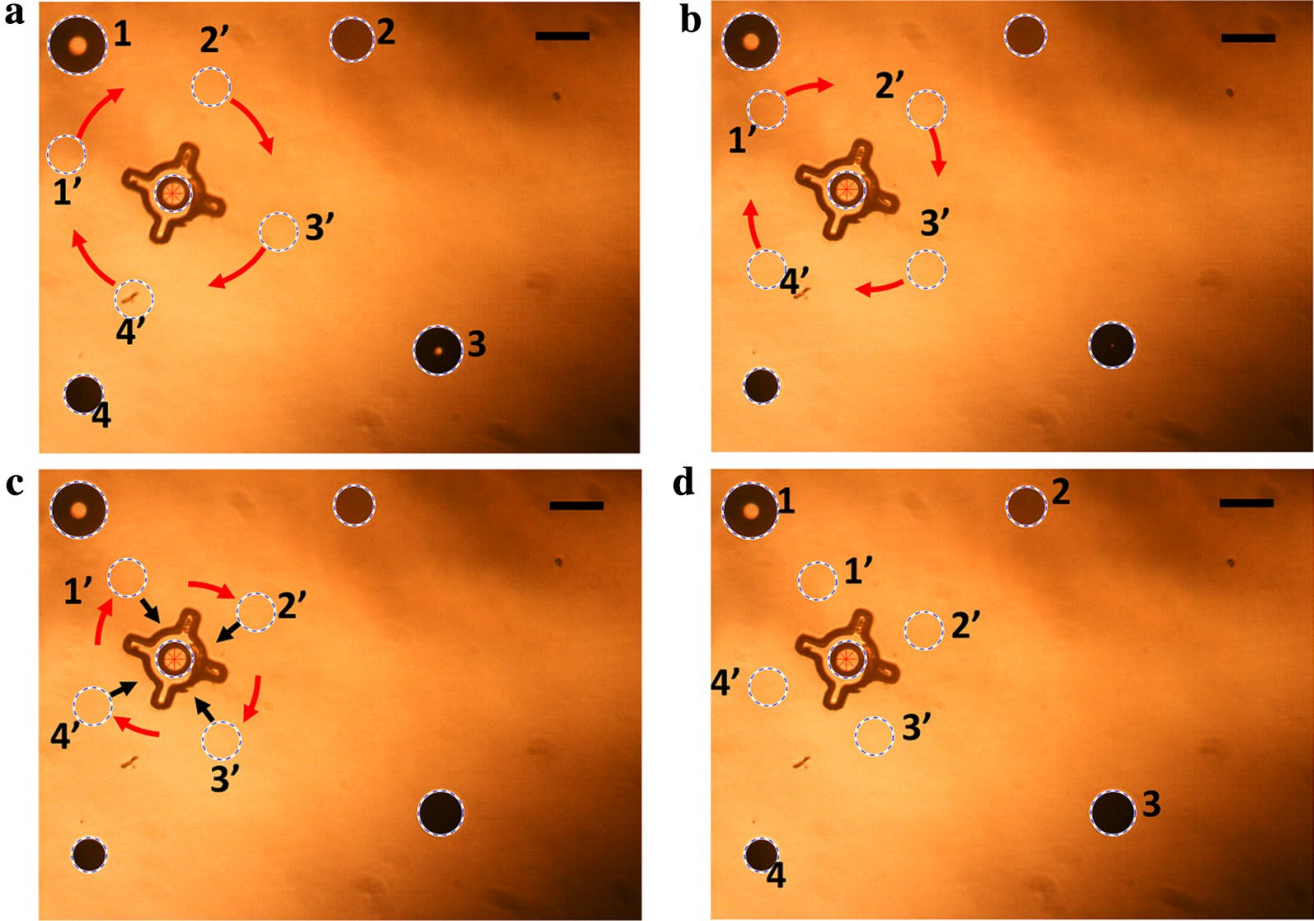
Determining the optimum caging location of the microrobots around a payload using the grasp-planning module. **a** The image-processing algorithm determines the location and size of the randomly located microrobots (labeled 1–4) and the micro-object. The path-planning algorithm calculates the caging positions, marked using dotted circles numbered 1′ to 4′. The initial caging location was set 307 μm away from the center of the micro-object. **b** The caging formation is rotated 10 degrees clockwise. **c** The formation is rotated another 10 degrees and contracted 51 μm using the grasp-planning module to position the microrobots between the arms of the star-shaped structure. **d** The final caging locations calculated by the grasp-planning algorithm are marked with dotted circles labeled 1′ to 4′. The size of Microrobots 1, 2, 3, and 4 is 156, 114, 126, and 101 μm in diameter. Scale bar: 150 μm

Initially, four OFB microrobots were generated at random locations around the micro-object (Fig. 4a) by momentarily increasing the optical power in each spot using the actuation block (Fig. 2). The feedback block then detects the location and size of the microrobots and the structure. The grasp-planning algorithm uses the location of the structure within the workspace to calculate the caging positions (1′, 2′, 3′, and 4′ in Fig. 4a) at user-defined equidistant locations around the micro-object. The grasp-planning algorithm allows the adjustment of the caging locations based on visual feedback and the shape of the object. In this experiment, the caging formation was rotated clockwise (Fig. 4a–c) to allow a better grasp of the micro-object. The final caging configuration (Fig. 4d) puts the microrobots in positions that will allow them to grasp in between the arms of the micro-object when the caging formation is contracted.

### Hybrid closed-loop actuation of multiple microrobots

The caging formation (1′, 2′, 3′, and 4′ in Fig. 4d) calculated by the grasp-planning algorithm of the feedback block sets the destination location for the individual microrobots. These positions were saved in a.mat file and subsequently loaded into the MathScript module of the LabVIEW actuation block. However, the caging locations require a transformation to match the coordinate system of the actuation block. Once the transformed final destinations of the microrobots are serially loaded into the MathScript module from the.mat file, the destination of each microrobot is mapped to the corresponding locations on the LabVIEW user interface. In this experiment, the microrobots numbered 1, 2, 3, and 4 were assigned the caging locations 1′, 2′, 3′, and 4′ (Fig. 4d) as their destinations in the actuation block.

**Fig. 5:**
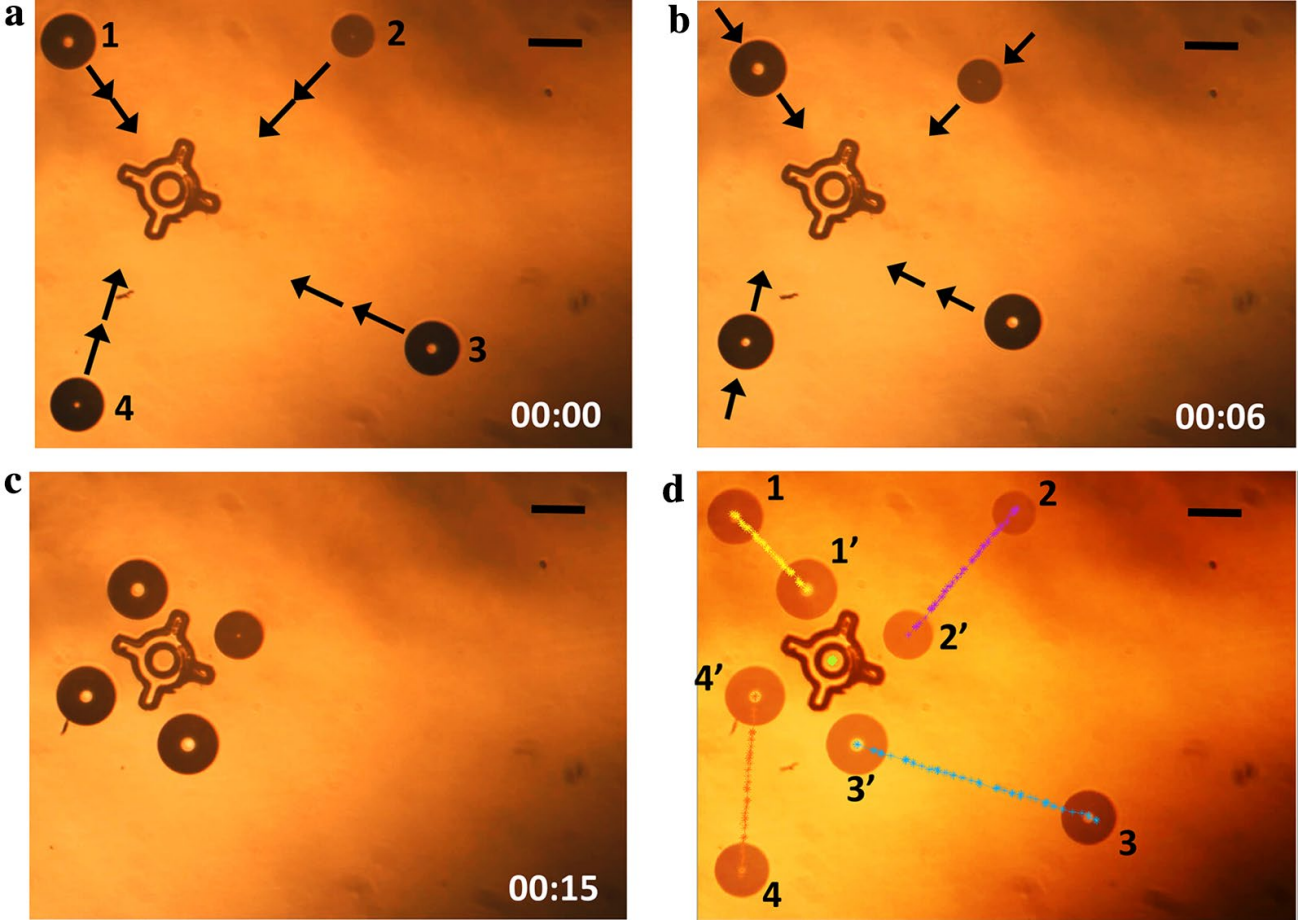
Automatic actuation of four OFB microrobots from their initial positions to the caging formation. **a** Microrobots are approaching to the caging formation. **b** Each microrobot is actuated simultaneously at different speeds. **c** Microrobots positioned around the micro-object. **d** Since the microrobots were actuated at different speeds, they all arrive at their caging positions at the same time, despite the differences in the length of the actuation paths. The average size of Microrobots 1, 2, 3, and 4 is 159, 132, 161, and 159 μm in diameter. Scale bar: 150 μm. Time format: minutes: seconds

Figure 5 shows the open-loop actuation of four microrobots from their initial positions to the caging locations. Microrobots 1, 2, 3, and 4 were simultaneously actuated at velocities of 19, 29.6, 44.1, and 31.83 μm/s, respectively, using the actuation block (Fig. 5a–c). Here, the simultaneous actuation of multiple microrobots with different speeds demonstrates a capability of the microrobot control system: parallel, uncoupled movement of microrobots along trajectories that vary in direction and distance traveled during the same actuation time. The microrobot actuation took 15 s (Additional file 2: Video S1). Figure 5d shows the path of each microrobot from its initial position to the caging location. Here, the simultaneous actuation of multiple microrobots with different speeds demonstrates a capability of the microrobot control system: parallel, uncoupled movement of microrobots along trajectories that vary in direction and distance traveled during the same actuation time.

**Fig. 6:**
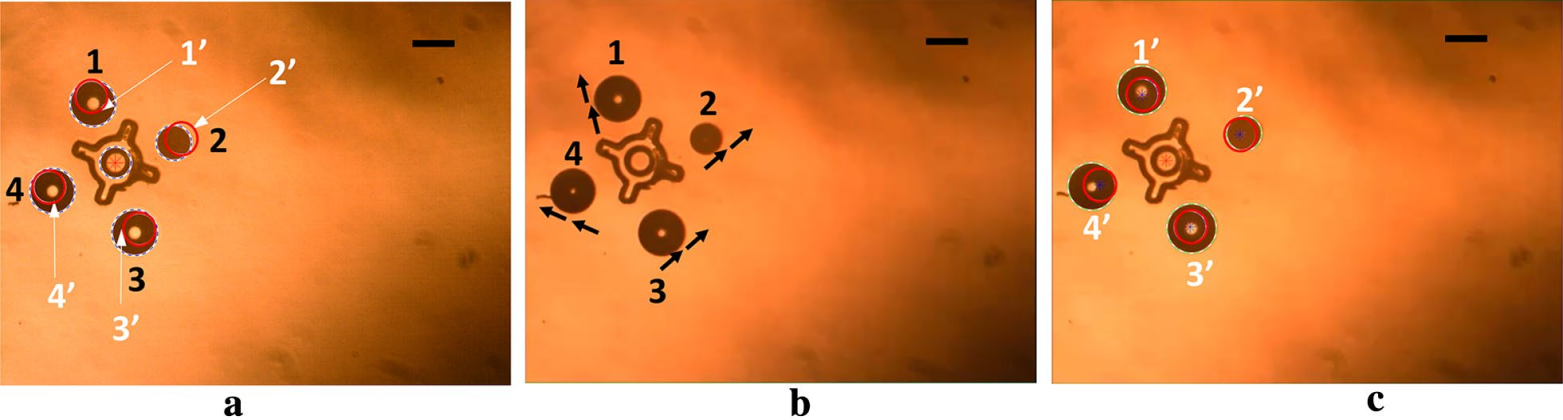
Closed-loop position updating of the microrobots at the caging formation. **a** Image captured by the feedback block showing the position error between the desired location of the microrobot (indicated by the red circle marked with white arrows) and the physical location of the microrobots (marked with the dotted white circle). **b** Microrobots are actuated to the new destination, minimizing the position error. **c** Microrobots actuated to the updated caging formation, resulting in reduced position error. The average size of Microrobots 1, 2, 3, and 4 is 149, 108, 150, and 144 μm in diameter. Scale bar: 150 μm

Figure 6 shows the OFB microrobots at the caging positions. The image-processing algorithm determined the locations of the microrobots and compared them to the caging locations calculated by the grasp-planning algorithm. Figure 6a shows the locations of Microrobots 1, 2, 3, and 4 as determined by the image-processing algorithm (white dotted circles), and the desired caging locations set by the grasp-planning algorithm (red circles). The feedback block calculates the error between the actual location of the microrobot and the desired caging position, and calculates the new microrobot destination to minimize the error. Microrobots 1, 2, 3, and 4 in Fig. 6a were 27, 24.7, 14.28, and 21.5 μm away from their desired caging positions, respectively. This information is passed to the MathScript node of the actuation block, which moves the microrobots to the new destinations (Fig. 6b). The microrobots were actuated to their new positions at speeds of 5.4, 4.94, 2.86, and 4.3 μm/s for Microrobots 1, 2, 3, and 4, respectively (Fig. 6b).

After updating the location using open-loop actuation, the physical location of the microrobot within the workspace was determined by the image-processing algorithm of the feedback loop, as shown in Fig. 6c. The red circles in Fig. 6c are the caging position set by the grasp-planning algorithm at the beginning of the actuation. In Fig. 6c, the Microrobots at 1′, 2′, 3′, and 4′ were 8, 12, 5, and 19 μm away from their desired locations, corresponding to a reduction in the position error of approximately 50%.

### Micromanipulation

**Fig. 7:**
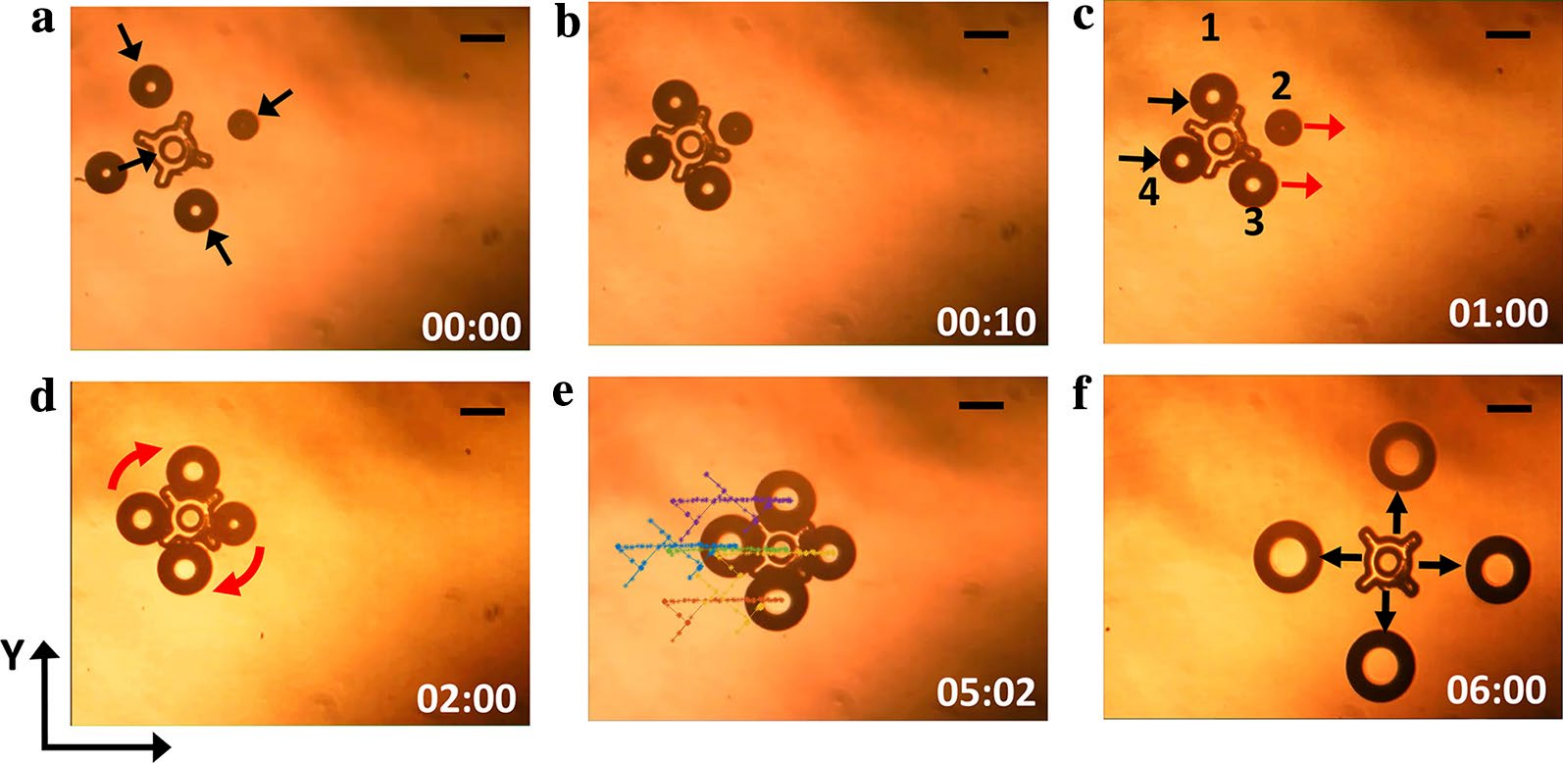
Micromanipulation by grasping using open-loop actuation. **a** Microrobots move from the initial caging formation to grasp the micro-object. **b** The microrobots have grasped the micro-object. **c** The formation of four microrobots is attempting to move the micro-object in the x-direction, but fail due to the object’s stiction with the substrate. Microrobots 2 and 3, marked by the red arrows, move right, leaving the micro-object behind. **d** A clockwise twisting motion was used to ease the stiction of the micro-object. **e** The micro-object was manipulated along a random trajectory at various speeds. **f** The payload is released within the workspace by expanding the microrobot formation using open-loop actuation. The average size of Microrobots 1, 2, 3, and 4 is 191, 167, 194, and 195 μm in diameter, respectively. Scale bar: 150 μm

The hybrid closed-loop vision-assisted control system allowed an accurate placement of a caging formation, as described above. Figure 7a shows the open-loop actuation of a microrobot matrix approaching a micro-object to grasp it for manipulation. The matrix of microrobots was manually controlled by user input to the actuation block. The object was grasped by contracting the microrobot formation at an average speed of 7.6 μm/s (Fig. 7b and Additional file 3: Video S2). After grasping, the microrobot formation attempted to transport the micro-object in the positive *x*-direction. However, the micro-object was stuck to the floor of the fluid chamber, resulting in the dislocation of the microrobots from their actuation patterns (Fig. 7c). This phenomenon is more obvious for Microrobots 2 and 3, marked with red arrows in Fig. 7c, as they were moved in the positive *x*-direction and left the micro-object behind.

To free the micro-object from the surface, the formation of the microrobots was rotated to create a torque on the object while maintaining a firm grasp (Fig. 7d). The twisting of the object helped to overcome its stiction, and the micro-object could then be transported. The micro-object was transported along various trajectories (Fig. 7e) and at various speeds up to approximately 90 μm/s (Additional file 4: Video S3). A graph of the planned trajectory and completed trajectory during the micro-object manipulation is included in Additional file 1: Figure S5. Upon completion of the micromanipulation, the micro-object was released by expanding the microrobot formation (Fig. 7f).

## Discussion

The hybrid closed-loop vision-based control of OFB microrobots with open-loop actuation has leveraged the functionality of two different platforms to perform the micromanipulation tasks. This integration of MATLAB and LabVIEW utilizes the hardware support and rapid configuration of LabVIEW and the advantages of MATLAB’s processing of complex image data. Aside from providing more precise manipulation, closed-loop position updates allow the use of standard macroscale robotic functions such as grasp planning, collision avoidance, and detection, grasping, and payload detection and delivery. Also, the hybrid control allows the detection of failed actuation of individual microrobots (Additional file 1: Figure. S6). Moreover, the OFB microrobot system, which is capable of the independent actuation of many microrobots, utilizes vision-based automatic actuation for the simultaneous participation of multiple microrobots in micromanipulation, as it is difficult for a human operator to control many microrobots. In this work, the OFB microrobot system enabled a team of microrobots to transport a large object, which is not possible with a single microrobot. This was quantified in previous work, as it was observed one or two microrobots could produce limited rotational movement of an SU-8 microstructure, but no translational movement. Three or four microrobots were necessary to translate the micro-object [40].

The closed-loop position updates increased the accuracy of the caging locations by 50% compared to one iteration of open-loop actuation. The results suggest that multiple iterations of closed-loop position updating may reduce the error further, but this needs more investigation. The causes of the position error during open-loop actuation can be broadly divided into two categories: system error and mechanical error. The system error is due to the spatial resolution of the SLM and any misalignment of the optical elements. The mechanical error is caused by the misalignment of the image coordinates compared to the LabVIEW user interface coordinates. This misalignment exists due to the mechanical adjustment of the camera position when attempting to match to the LabVIEW coordinates. The software-defined coordinates in LabVIEW were considered the ideal coordinates, and the camera was adjusted by hand such that a single pixel on both the MATLAB image-processing module and the LabVIEW actuation module had the same dimensions. Despite the careful adjustment, an error of approximately 6–8 pixels was present; this was quantified by taking multiple measurements of a stationary micro-object.

The closed-loop control system helps with the system error, but is unable to correct for the mechanical error. The average error calculated during open-loop actuation (Fig. 5) was 21.87 μm per microrobot. The closed-loop position update reduced the average error to 11 μm per microrobot.

The positioning tolerance of the micromanipulation varies with the size of the objects under manipulation and the type of manipulation. For example, stable caging requires the microrobots to be placed at a distance smaller than the payload size from each other [41, 42]. The caging of the 300-μm-diameter star-shaped micro-object using 130-μm-diameter microrobots required the microrobots to be placed less than 300 μm from each other for stable trapping and at least 130 µm (one body length) apart to avoid merging of the bubble microrobots. Thus, the calculated microrobot separation of 280 μm with ± 10 μm position tolerance satisfies these conditions. The hybrid closed-loop control system was able to reduce the average position error from 21.9 to 11 μm, satisfying the position tolerance for this micromanipulation task.

The image acquisition, object detection, and path planning in MATLAB take approximately 1.6 s to compute while running on a PC with an AMD Phenom II × 6 3.31-GHz processor and 16 GB RAM. The object locations and path planning data calculated by the feedback block (Fig. 2) are saved in a.mat file. A MathScript node included in the actuation block in LabVIEW reads the data from the.mat file and sends it to the sequence generator for actuating the microrobots along the planned path. The feedback block runs in MATLAB and the actuation block runs in LabVIEW, so the operator needs to manually enter the.mat file location in the MathScript node and click the run button in the actuation block. This operation takes approximately 5 s, so there is a total delay of approximately 6.5 s between the image acquisition and the microrobot actuation.

The image feedback of the proposed hybrid control system can detect more than four particles at once; it can detect as many as distinct objects that can fit in the camera field of view. The control algorithm is also not limited to four particles; it can set destinations and waypoints for a number of objects detected in the workspace. The open-loop control part of the hybrid system has been demonstrated to control an array of 50 OFB microrobots [40], and this system can control at least the same amount of microrobots at once.

In this work, the vision-based closed-loop position update was executed at the waypoints for single microrobot actuation and at the caging locations for multiple microrobot actuations, instead of iteratively after each camera video frame. The closed-loop position update was not implemented after each frame since the position errors per frame were usually less than one micron. Moreover, the processing time for the image analysis of the high-resolution image (1600 px by 1200) at each frame would increase the overhead on the overall computation process and time.

## Conclusions

A hybrid closed-loop vision-assisted control system was developed in MATLAB and LabVIEW to control multiple OFB microrobots automatically. The control system was used to demonstrate open-loop actuation of a single microrobot and simultaneous actuation of multiple microrobots along with closed-loop position updates. A grasp-planning algorithm was also developed in MATLAB and utilized to calculate the precise locations for grasping a micro-object using a team of four microrobots. The position of each microrobot and the micro-object under manipulation was detected by the closed-loop feedback module to minimize the error between the physical location calculated by the image-processing algorithm and the intended destination. This closed-loop actuation allows automatic and simultaneous actuation of multiple microrobots for micromanipulation with precise positioning, beyond the capacity of a human operator. However, this hybrid control scheme requires certain human operators to switch between the applications (MATLAB and LabVIEW). In the future, this system can be upgraded to use image processing based on the seamless integration of LabVIEW and MATLAB, allowing minimum user interaction.

## Additional files

**Additional file 1.** Supplementary information.

**Additional file 2: Video S1.** Automatic actuation of four OFB microrobots into the caging formation.

**Additional file 3: Video S2.** Grasping the micro-object.

**Additional file 4: Video S3.** Micro-object manipulation.
