# Supplementary material for: Vision-assisted micromanipulation using closed-loop actuation of multiple microrobots
Source: Robotics Biomim. 2017 Oct 30;4(1):7. doi: 10.1186/s40638-017-0064-4 (PMC5662703; doi:10.1186/s40638-017-0064-4)
Supplement: Supplementary file 1 — Additional file 1. Supplementary information. [file 40638_2017_64_MOESM1_ESM.docx]

**Supplementary Information to**

Vision-Assisted Micromanipulation Using Closed-Loop Actuation of Multiple Microrobots

M Arifur Rahman, Noboru Takahashi, Kawai F. Siliga, Nigel K. Ng, Zhidong Wang and Aaron T. Ohta^.^

**1. Collision-Free Path Determination**

The path planning module of the hybrid control system is capable of determining a collision-free path to a destination. Figure S5 demonstrates the calculation of a collision-free path for Microrobot 1. The feedback block detected the locations and shapes of the objects in the workspace, including the inner circular feature of the micro-object (blue circle in Fig. S5). The destination location of Microrobot 1 is marked by the black dotted circle; the shortest path from the initial location Microrobot 1 to the destination is obstructed by the micro-object. The path-planning algorithm takes the object locations, the destination of Microrobot 1 as entered by the operator, and the minimum collision-free distance (MCD) set by the operator as inputs for calculating the collision-free path. The MCD was set to 2 times the radius of the blue circle marking the micro-object. The collision-free path was calculated from the current location towards the destination along a linear trajectory until the microrobot reaches the MCD (P1 in Fig. S5). At P1 the path continues along a curved path towards the destination, maintaining the MCD from the center of the obstacles until it reaches P2. From P2 the path runs on the shortest straight line towards the destination, marked with a black dotted circle.

**
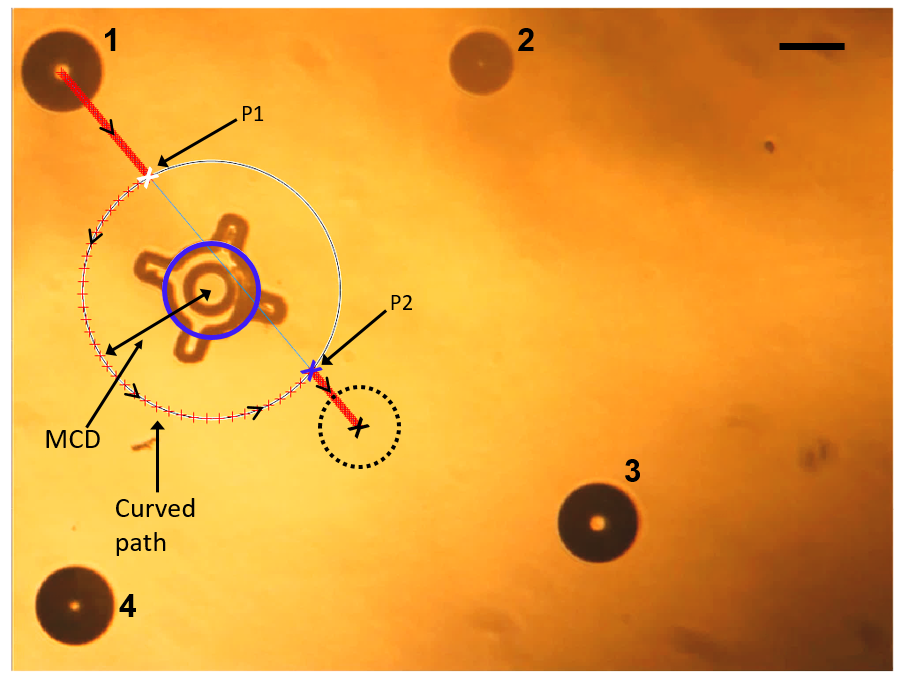
**

**Figure S4.** Collision-free path calculation by the path-planning algorithm. The path was calculated for Microrobot 1 from its current position to the destination marked with dotted black circle, while avoiding the obstacle marked with a blue circle. The calculated path consists of linear paths and a curved path to avoid the obstacle.

**2. Movement Accuracy During Micromanipulation**

A micro-object was manipulated in different directions along random trajectory by grasping with four microrobots (Figure 7e and Supplementary Video S3). Figure S6 shows the trajectory of all four microrobots, with measured locations marked with asterisks. All four microrobots were grasping the micro-object, resulting in similar trajectories. The planned trajectory of Microrobot 2 (Figure 7c & e) is plotted in Figure S6 using a black dotted line. The error between the planned (black dotted line) and the actual (yellow line) trajectory has a maximum approximately 15 μm, suggesting that closed-loop position updates at certain waypoints is sufficient to maintain an acceptable error range on the order of 10 μm.


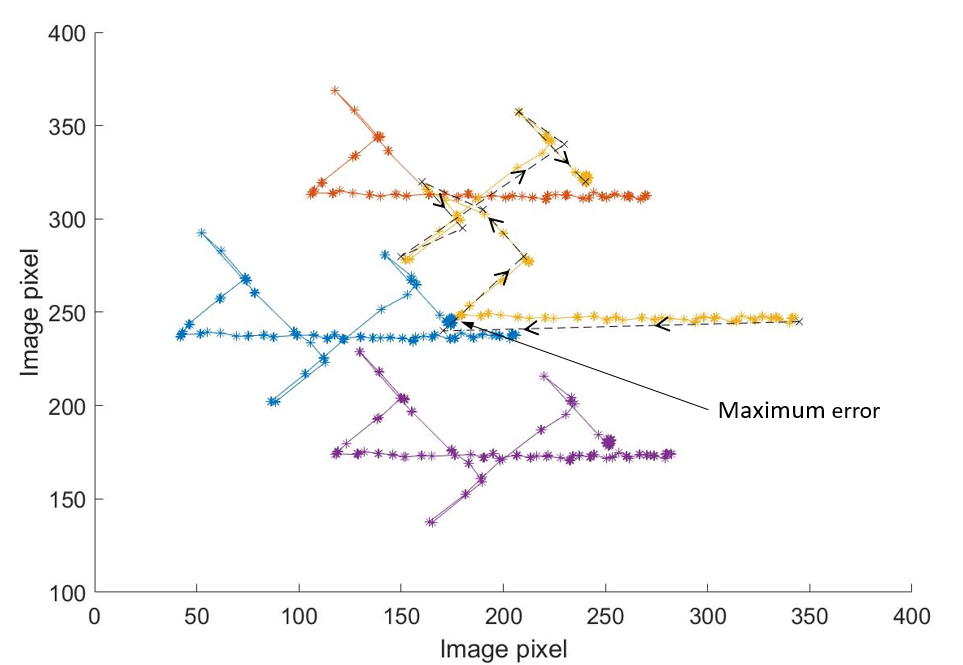


**Figure S5.** The measured trajectory of four microrobots actuating grasping a micro-object (lines with asterisks) and the planned trajectory of one of the microrobots (black dotted line).

**3. Microrobot Actuation Error Detection and Correction**

The hybrid control system changes the position of the laser spots with the knowledge of microrobot location. In contrast, open-loop control moves the optical pattern even if the microrobot under actuation does not follow the location of the laser spot. This is demonstrated in Figure S4. An OFB microrobot was intended to be actuated from its initial position to the location marked by a dotted black circle, 1200 µm away (Fig. S4a). The actuating laser spot was translated at approximately 400 µm/s, but the microrobot was unable to move at this speed (Fig. 4b). The image-processing function of the feedback block detected that the microrobot failed to follow the optical spot, and identified the current location of the microrobot (Fig. 4b). The laser spot was translated back to the location of the microrobot, and another attempt was made to actuate the microrobot to the destination at a lower translational velocity of approximately 60 µm/s, resulting in successful movement to the destination (Fig. 4c). This type of actuation error may occur for various reasons, such as a high translational velocity of the optical spot, insufficient power at the actuating beam, and collision of the microrobot with obstacles. This hybrid closed-loop control system can detect and recover from these actuation errors.


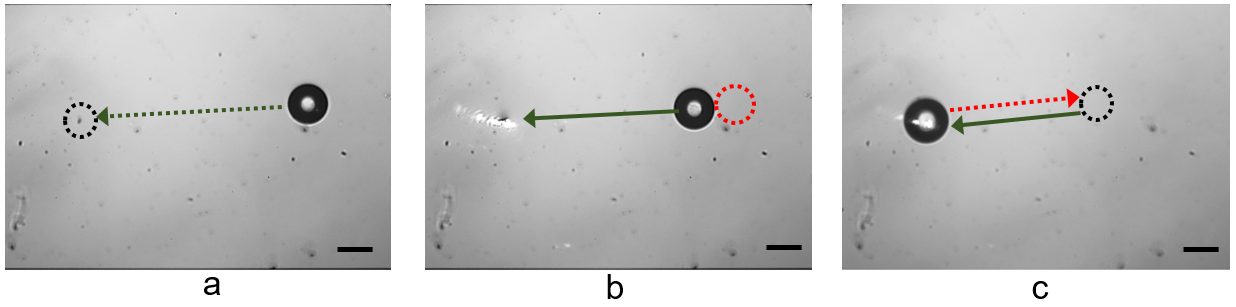


**Figure S6.** Microrobot actuation error detection and correction using the hybrid closed-loop control system. (a) The microrobot was to be actuated to the destination marked with the black dotted circle. (b) The laser spot was translated at high speed to the target location (following the path of the green arrow), leaving the microrobot behind. The microrobot was actuated 187 µm from its initial position (dotted red circle) in the direction of the laser movement, but failed to follow any further. (c) The feedback block detected the microrobot position and passed it to the actuation block. The actuation block translated the laser beam towards the microrobot, as indicated by the red dotted arrow. Then the microrobot was actuated to the destination a a lower speed, along the path indicated by the green arrow.
